# The Reproductive Ecology of Industrial Societies, Part II: The Association between Wealth and Fertility

**DOI:** 10.1007/s12110-016-9272-9

**Published:** 2016-09-26

**Authors:** Gert Stulp, Rebecca Sear, Susan B. Schaffnit, Melinda C. Mills, Louise Barrett

**Affiliations:** 1 Department of Population Health, London School of Hygiene & Tropical Medicine, Keppel Street, London, WC1E 7HT UK; 2 Department of Sociology, University of Groningen / Inter-university Center for Social Science Theory and Methodology (ICS), Grote Rozenstraat 31, 9712 TG Groningen, The Netherlands; 3 Department of Sociology and Nuffield College, University of Oxford, Manor Road, Oxford, OX1 3UQ UK; 4 Department of Psychology, University of Lethbridge, Lethbridge, AB T1K 3M4 Canada

**Keywords:** Wealth, Income, Fertility, NLSY79, Secondary database

## Abstract

Studies of the association between wealth and fertility in industrial populations have a rich history in the evolutionary literature, and they have been used to argue both for and against a behavioral ecological approach to explaining human variability. We consider that there are strong arguments in favor of measuring fertility (and proxies thereof) in industrial populations, not least because of the wide availability of large-scale secondary databases. Such data sources bring challenges as well as advantages, however. The purpose of this article is to illustrate these by examining the association between wealth and reproductive success in the United States, using the National Longitudinal Study of Youth 1979. We conduct a broad-based exploratory analysis of the relationship between wealth and fertility, employing both cross-sectional and longitudinal approaches, and multiple measures of both wealth (income and net worth) and fertility (lifetime reproductive success and transitions to first, second and third births). We highlight the kinds of decisions that have to be made regarding sample selection, along with the selection and construction of explanatory variables and control measures. Based on our analyses, we find a positive effect of both income and net worth on fertility for men, which is more pronounced for white men and for transitions to first and second births. Income tends to have a negative effect on fertility for women, while net worth is more likely to positively predict fertility. Different reproductive strategies among different groups within the same population highlight the complexity of the reproductive ecology of industrial societies. These results differ in a number of respects from other analyses using the same database. We suggest this reflects the impossibility of producing a definitive analysis, rather than a failure to identify the “correct” analytical strategy. Finally, we discuss how these findings inform us about (mal)adaptive decision-making.

**Electronic supplementary material:**

The online version of this article (doi:10.1007/s12110-016-9272-9) contains supplementary material, which is available to authorized users.

Evolutionary analyses of fertility^1^ behavior among industrialized populations have increased in both frequency and prominence in recent years. This reflects both an on-going interest in trying to understand patterns of fertility decline within an evolutionary framework (e.g., Sear et al. 2016), as well as greater recognition of the potential that large-scale population surveys hold for testing evolutionarily relevant questions (Nettle et al. 2013). Some advocates of an evolutionary approach, however, argue that the study of modern fertility behavior is rather uninformative and should be foregone in favor of studying psychological processes, under the assumption that cultural change has been too rapid for genetic evolution to keep pace and has resulted in a mismatch between our evolved adaptations and modern-day industrial environments. In our companion paper (Stulp et al. 2016), we take issue with this assessment, arguing that the measurement of fertility continues to matter because (1) we have to test the assumption that natural selection has been negligible in recent times, not simply regard it as axiomatic; (2) only measurements of fitness components, such as fertility, can provide evidence for a maladaptive mismatch between ancestral environments and the present day; and (3) if our current behavior does turn out to be maladaptive, patterns of fertility within and between industrial populations can provide insight into the psychological mechanisms and environmental conditions that result in a failure to maximize fitness. In addition, in that paper we considered both the advantages and limitations of large-scale secondary databases in tackling the question of the (mal)adaptiveness of modern-day fertility behavior.

Here, we expand on the central points of our companion paper by presenting an example that walks through the methodological and analytical challenges presented by the National Longitudinal Survey of Youth 1979 (NLSY79), a large US database, as we explore the relationship between fertility behavior and wealth (in the form of income and assets). Wealth, as a measure of access to resources, is an evolutionarily relevant variable, and studies of the association between wealth and fertility have a long history in economics (e.g., Becker 1960; Easterlin 1975) and in the evolutionary social sciences (e.g., Pérusse 1993; Turke 1989; Vining 1986; see Hopcroft 2006; Nettle and Pollet 2008; Stulp and Barrett 2016a for reviews). In particular, findings of a negative association between wealth and fertility have been used as evidence to suggest humans are maladapted to the conditions of industrialized societies (e.g., Tooby and Cosmides 2015; Vining 1986), but other studies suggest the relationship is more complex than these analyses suggest, and initial findings may reflect the kinds of data used and the manner in which they are analyzed (e.g., Stulp and Barrett 2016a). Thus, our use of the wealth-fertility relationship as a case study is well suited to exploring the challenges of secondary databases. In addition, we can use the results of our analysis to address another point made in our companion paper—namely, that the study of fertility behavior in industrial societies can shed light on the mechanisms that produce these patterns and help explain when and why people fail to behave in a fitness-maximizing fashion. However, our analyses are not intended to be a comprehensive exploration of the association between wealth and fertility, nor do we aim to provide a full explanation of (mal)adaptive behavior. Rather we focus on those elements that best illustrate some of the conceptual points made in our companion paper.

## The National Longitudinal Survey of Youth 1979

The precise research question we ask here is: Are different forms of material wealth—income (or earnings) and net worth (or assets)—associated with fertility outcomes? To answer this, we first provide some descriptive analyses of fertility (adjusted for mortality) and wealth patterns in the NSLY79 and then present cross-sectional analyses that test for an association between these forms of wealth and lifetime reproductive success (the number of surviving children). We then use a longitudinal analysis—which is more appropriate for establishing the direction of causality—to examine whether wealth influences the initial transition to parenthood and to the production of a second and third child. This strategy is less commonly pursued in the evolutionary literature, possibly because of a focus on lifetime reproductive success as a proxy for fitness. As we proceed, we discuss the methodological and analytical issues that researchers face when attempting such analyses, including sample selection, the existence of confounding variables, the heterogeneity of large samples, assessing data quality, and identifying various forms of bias.

Although we consider a detailed methods section to be the most important part of any paper, given that our main aim is to highlight the difficulties that arise when conducting secondary analyses, we provide only the most relevant aspects of our analyses here. We fully recognize that this seems to be in conflict with our plea for clarity in terms of sample selection and the decision to include and exclude variables, but in order to keep the paper to a bearable length, we decided to place the full details of the sample, variables, analyses, and imputations in the ESM. See ESM Tables 1 and 2 for descriptive statistics. All analyses were performed in R (R Development Core Team 2008), including the use of the lme4 package. Graphics were produced using ggplot2 (Wickham 2009). We use *p* values to indicate whether there is strong or weak evidence for an association, but we refrain from assessing relationships as “significant” to avoid assessments in terms of arbitrary cutoffs.

### Sample Selection and Researcher Degrees of Freedom

Large, complex datasets contain an enormous array of data and therefore present researchers with an equivalently large number of “degrees of freedom” (i.e., the choices made about which variables to analyze, and how). Consequently, sample selection and the choice of (confounding) variables to include in the analyses are the first decisions that have to be made, and they are some of the most important; such decisions can have a profound influence on the outcome of an analysis (Silberzahn and Uhlmann 2015). It is crucial, therefore, to explain how and why such decisions are made. As such, we begin by accounting for our decision-making process with respect to sample selection. We also note where these choices differ from the decisions made by other studies using the same database and tackling similar research questions. We then deal with the issue of heterogeneity that is often found in large-scale databases, using the ethnicity of respondents as our example, and whether there are differences in reproductive measures and partnership formation that need to be accounted for.

Our study uses the National Longitudinal Survey of Youth 1979 (NLSY79), which follows the lives of 12,686 individuals (6283 females) born between 1957 and 1964. Respondents were first interviewed in 1979, when their ages varied between 14 and 22. Respondents have been interviewed subsequently every year up until 1994, and every two years after that. The last round of interviews took place in 2012, when the respondents were between 47 and 56 years old.

The NLSY79 divides ethnicity into three large categories: non-black/non-Hispanic (*N* = 7150), black (*N* = 3174), and Hispanic or Latino (*N* = 2002). Moreover, the study consists of three different subsamples: (*i*) a cross-sectional sample of respondents (*N* = 6111) designed to represent the noninstitutionalized civilian segment of the population; (*ii*) a supplementary sample of civilian Hispanic or Latino, black, and economically disadvantaged nonblack/non-Hispanic respondents (*N* = 5925), and (*iii*) a sample designed to represent the population serving in the US military (*N* = 1280). We decided to drop the military sample because funding constraints severely limited the sample size from 1984 onwards (after which only 201 respondents remained). We also excluded the economically disadvantaged nonblack/non-Hispanic respondents, because these individuals were no longer followed after 1990, and also because their inclusion would have biased our sample toward poorer individuals.

The sampling design of the survey involved selecting every eligible person of a certain age within the household. Thus, family members and spouses of the main respondents living in the household were also included in the sample. To avoid issues of non-independence and pseudo-replication that can arise from such sampling, we decided to include data only from the first selected respondent. We also excluded respondents who had served time in prison because incarceration obviously hinders reproductive decision-making and income is not generated.

These decisions differ from those of a recent study by (Hopcroft 2014), using the same data to answer a similar question. She selected a different subsample (dropping, for instance, all of the supplementary samples), plus multiple individuals from the same household were included in the analyses, along with individuals who had served time in prison. Another recent study chose similar subsamples to us but again included multiple individuals per household (Breen and Chung 2015). Differences in study outcomes can therefore arise from decisions made at the very beginning of a study, before any analytical procedure has even been attempted (see the studies of Sear et al. 2004 and Courtiol et al. 2013 for a striking example in which the selection of a different subsample and analytical strategy led to exactly opposite conclusions; see Stulp and Barrett 2016b for possible explanation).

## Descriptive Analysis of Reproductive Patterns

**Fig. 1:**
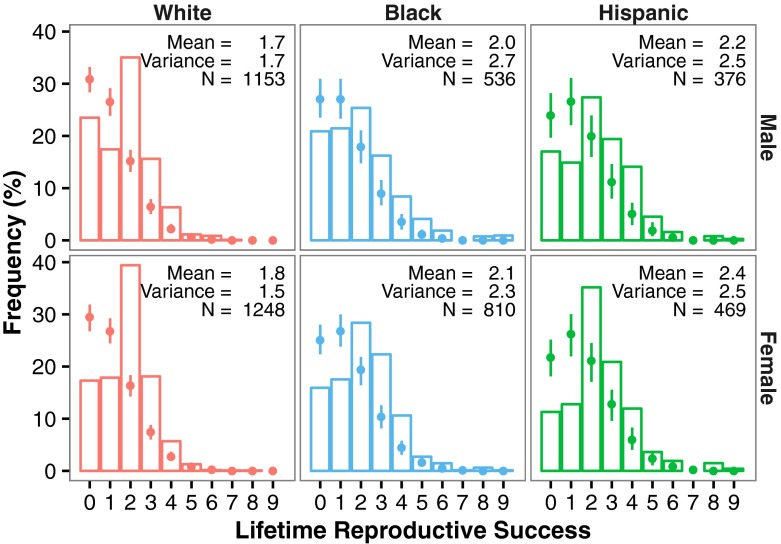
Frequency distribution of lifetime reproductive success (number of surviving children) for white, black, and Hispanic men and women. Dot and error bars reflect median and 95% range of 1000 random Poisson simulations of similar sample size and mean

The first step in our analysis was to describe overall patterns in (proxies of) fertility. These patterns can, by themselves, begin to offer an insight into reproductive decision-making. We start by showing, in Fig. 1, the frequency distribution of the number of surviving children, or lifetime reproductive success (LRS; by sex and ethnic group). For these analyses, we chose to use the lifetime number of surviving children because this is a better proxy for fitness than the number of children ever born (i.e., fertility); this is particularly relevant because increased wealth has been shown to have a protective effect on child survival even in low-mortality populations (Remes et al. 2011).

### Retention and Response Bias

When measuring LRS, we restricted our sample to those individuals aged 45 and over since probabilities of birth are low beyond this age (only 0.3% of women and 3% of men in our sample had their last birth over the age of 45). This restriction means that our sample of respondents contained only those people who had participated in follow-up interviews for at least twenty years. This is likely to introduce a bias because the likelihood of dropout over time is not random (e.g., Rendall et al. 1999; Watson and Wooden 2006)—the people retained by the sample are likely to share certain characteristics that are not shared by those who dropped out. Having said this, the NLSY79 has remarkably high rates of retention (Zagorsky 1999) and is seen as the “gold standard” for longitudinal studies (Randall 2005). This kind of retention bias may therefore be a relatively minor concern. In addition, our longitudinal analyses will account for right-censoring (i.e., the fact that younger individuals will not have completed their reproductive life spans or that people may exit the study before the final wave of data collection).

These descriptive analyses also raise a further methodological point and highlight a well-known limitation of secondary databases. It is apparent that women report more surviving children than men (Fig. 1). Controlling for ethnicity, women report 8% more ever-born children and 7% more surviving children than men (a similar magnitude for this sex difference is observed in a different sample from the US; Stulp et al. 2012). Given that every child must have both a mother and a father, this indicates a bias. One possibility is that, because of their longer (potential) reproductive life spans, men continue to produce offspring at much older ages, but those children are not captured by our sample because, by necessity, we are only able to follow the men into their mid-fifties (i.e., some of these men may continue to have more children). Although, of course, this question can be answered following future waves of data collection, it remains speculative at present. In addition, some of the women in the sample may be reproducing with much older men, but the men themselves do not form part of the sample population. The inclusion of these “missing” births and “missing” men could narrow the sex difference in this measure of fertility.

Having said this, given that births at older ages occur only sporadically (Stulp et al. 2015), the differences in the number of children reported by men and women are more reasonably explained by reporting biases and nonrandom dropout (Rendall et al. 1999): men are more likely to underreport births from previous marriages and, in particular, extramarital births, and previously married men are likely to be underrepresented in the sample. Men may also underreport the number of children they have fathered through simple ignorance of paternity, whereas this is unlikely to affect female respondents. Given these problems, not all of which can be solved satisfactorily, it is not surprising that demographers have typically ignored male fertility (Becker 1996; Greene and Biddlecom 2000; Watkins 1993). In terms of our subsequent analyses, it is evident that the sexes must be treated separately (something likely to be true in any case, given that different processes may guide fertility decisions in men and women), and we should be particularly cautious when interpreting the results for men.

### Simulation Choices and Population Heterogeneity in Reproductive Strategies

To get some feel for whether observed patterns of LRS show evidence for variation in reproductive rates across individuals, we compared the observed distribution of the number of surviving children to that expected on the basis of a Poisson distribution with a similar mean (and with variance equal to the mean), where all individuals have a similar reproductive rate. We chose the Poisson model as our null model for two reasons: (1) it is a rather parsimonious distribution in which only one parameter has to be specified (the mean of the distribution), and (2) previous studies have used exactly this modeling strategy, and we wished to ensure comparability with these findings (Hruschka and Burger 2016; Morita et al. 2012, 2015). Whether the Poisson model really is a good null model is debatable (given the large discrepancies of the observed and actual distributions, one could argue that it is not), and obviously, we could have chosen different distributions (e.g., negative binomial, zero-inflated, or hurdle models). Nevertheless, the use of the Poisson is justified by our desire for a parsimonious simulation strategy that would enable a comparison with previous work. We also decided to present separate analyses for each ethnicity in case there are differences in reproductive rates and strategies that map onto this form of heterogeneity in the database.

We therefore generated 1000 simulations of Poisson distributions and determined the frequency of specific fertility outcomes (from no births to a maximum of nine) across all simulations. We then calculated a median value and 95% range across all 1000 simulations. So, for example, a median value of 20%, with a 95% range of 15–25%, for a fertility of 2 indicates that about 20% of families should have two births if the distribution is the outcome of a Poisson process, and that in 95% of all cases this value lies between 15% and 25%. If the observed values fall outside the calculated 95% range, this suggests that fertility outcomes in this sample have deviated from a situation in which all individuals reproduce at a similar rate. Our simulations revealed clear evidence that this was the case: in particular, the likelihood of remaining childless or having only one child were both much lower than expected, whereas families with two and, to a lesser extent, three children occurred at much higher frequencies than expected.

These patterns hold across all ethnicities, although they are particularly striking among white men and women, among whom there are approximately twice as many two-child families as expected (Fig. 1; only the first 9 births are plotted). White men and, in particular, white women also display very low variation in fertility (i.e., in the case of women the variation is lower than the mean, indicating underdispersion), showing that people behave more similarly than expected if everyone were to possess a similar reproductive rate.

This similarity in behavior (in particular the low number of one-child families and the high number of two-child families) is highly suggestive of a fertility norm. Preferences for two-child families (Carey and Lopreato 1995; Morita et al. 2015; Sobotka and Beaujouan 2014) and a disinclination to produce an only child are well established (Blake 1974) and fit well with these findings. In addition, these patterns show that more people reproduce and have at least one child than would be expected on the basis of a Poisson process with such a low mean. These findings therefore help frame and contextualize our subsequent analyses concerning wealth: given such low variation in fertility, particularly among whites, resources are unlikely to have the same magnitude of effect as that observed among nonindustrial populations (Nettle and Pollet 2008).

**Fig. 2:**
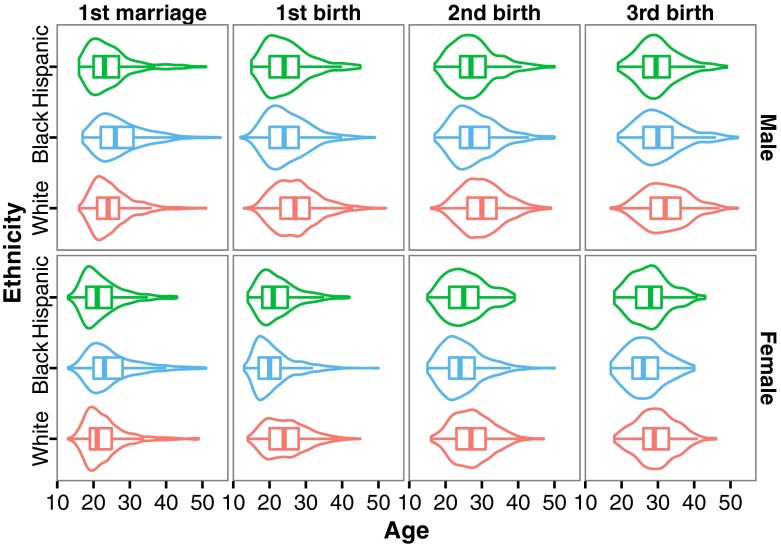
Box- and violin-plots of age at first marriage and age at the first three births for white, black, and Hispanic men and women

In addition, the differences between white and the other ethnic groups are sufficient to warrant the interpretation that these groups have different reproductive strategies, and hence that wealth may have a differential influence across these groups. Such intuitions are borne out by patterns in age at marriage and age at the first three births (Fig. 2): Hispanic individuals marry and give birth at young ages, and they reach the highest overall LRS of all three groups. White individuals marry similarly young (and have highest rates of getting married; see ESM Tables 1 and 2) yet give birth at older ages and achieve lower overall LRS. Black individuals marry latest and also have the lowest rates of marriage, yet they have their children at ages similar to Hispanics and reach similarly high levels of fertility (Fig. 2). These results, as well as previous research, suggest that different ethnic groups may indeed follow different reproductive strategies, with variation seen in total fertility/LRS (although these differences are decreasing), the timing of childbearing, the relational context of having children (e.g., married or not), and in the frequencies of intended and unintended births (related to contraceptive use; see Sweeney and Raley 2014 for review). At the very minimum, then, these patterns suggest that it is important to account for ethnicity in this sample when examining fertility outcomes. Thus, as with sex, we chose to analyze the different ethnicities separately in all our analyses.

## Descriptive Analysis of Wealth

### The Many Measures of Wealth: Income versus Net Worth

Having described patterns of fertility, we then turned our attention to how we would measure wealth in our analyses. Wealth has many meanings, and different measures of wealth could influence fertility outcomes in different ways. This, then, is another reason why we selected the NLSY79 cohort for our analyses: it represents a rich source of information on economic resources (Zagorsky 1999). Each wave includes detailed questions on various sources of income, as well as information on assets and debt. More specifically, assets minus debt is calculated to construct a variable that represents an individual’s “net worth.” This “net worth” is what economists have in mind when they talk about “wealth” (e.g., Emmons and Noeth 2015). In what follows, we refer to this as “net worth” because the term “wealth” has a broader meaning in human behavioral ecology (e.g., Borgerhoff Mulder and Beheim 2011), and we do not wish to confuse the two. Although income feeds into net worth (Semyonov and Lewin-Epstein 2013), they are not necessarily strongly related: for a given income level, there can be stark variations in net worth (Braveman et al. 2013; Jez 2014; Keister 2000, 2003; ESM Figure 3). Indeed, inequality in net worth is much more pronounced than inequality in earnings (Semyonov and Lewin-Epstein 2013).

A high net worth is considered more beneficial to individuals than receiving a certain level of income because, among other things, it increases financial stability by providing a buffer for emergencies, it can be passed on to subsequent generations, and it can be invested in ways that generate further wealth (Jez 2014; Keister 2003). Importantly for our purposes here, income and net worth (sometimes also referred to as the flow and stock of resources, respectively) can have different effects on particular kinds of outcome measures, such as health and education (Braveman et al. 2013; Jez 2014). It therefore seems reasonable to test whether the same is true of reproductive outcomes, especially as net worth is not often measured or included in studies of health, education, or fertility (Braveman et al. 2013; Jez 2014; Stulp and Barrett 2016a). This is also true of most studies in the human evolutionary sciences. Here, we examine the effects of both income and net worth on fertility decisions using both cross-sectional and longitudinal analyses.

### Variable Construction

For our analyses, we constructed a value of respondent income on the basis of income generated from the labor market—in other words, income from wages, salaries, and tips, from business and farm work, and from military income (sometimes referred to as “earnings”; see Breen and Chung 2015 for a similar strategy). Note that this excludes income from other sources, such as alimony, child support, unemployment compensation, or food stamps, because (*a*) they were not consistently measured across years and (*b*) the amount of money received from some of these sources depends on household characteristics, including the number of dependent children. Our measure therefore contrasts with a previous study that used income from wages only (Hopcroft 2014). In addition, we used income data from 1983 onward only, as this was the year in which questionnaires were standardized and identical across all respondents.

For the years 1985–1990, 1992–2000, 2004, 2008, and 2012, respondents were asked questions about their assets as well as income. In addition to providing information on “raw assets,” these data are represented in the database as a constructed variable of “net worth.” Here, for each survey year, the total value of assets a respondent possessed (including the worth of their home, cash savings, stock and bond portfolios, estate, business, and automobile assets, along with retirement and other saving plans) has been subtracted from their debt (including mortgage debt, property, business and automobile debt). Net worth can therefore take a negative value for those with more debts than assets. This newly constructed variable was checked for inconsistencies and missing data were imputed (details in the ESM). Net worth thus provides us with a measure of economic “wealth” and illustrates how the rich data characteristic of large-scale databases can be exploited to generate measures that are meaningful and directly relevant to the target question.

### Top-Coding, Response Bias, Retention Bias, and Reliability of Measures

For reasons of confidentiality, all income variables in the NLSY79 are top-coded (i.e., data points above an upper bound are censored), and in varying ways (Zagorsky 1999; e.g., the highest 2% of incomes are given the average value of that 2%). This artificial truncation of the wealth continuum obviously presents a limitation on any analysis performed using these data. Since 2% is a very small fraction, however, and since we are not specifically interested in the very wealthy, but in the entire wealth distribution, this limitation is unlikely to distort our findings, and we decided to include the top-coded values in our analyses. Nevertheless, top-coding is one of the reasons why income variables need to be transformed in order to allow comparability across survey years and respondent age (see below). Other studies using the same dataset have variously discarded the top-coded individuals (Breen and Chung 2015; Zagorsky 1999) or retained the top-coded values (Hopcroft 2014) during analyses.

Another problem pertinent to our analyses (and to secondary databases more generally) is that of response bias. This can be a particular problem when dealing with sensitive issues, including measures of net worth and income (Ross and Reynolds 1996). Zagorsky’s (1999) detailed investigation of the income and assets data in the NLSY79, however, revealed that only a very small proportion of individuals either refused to answer or did not know the specific value of their assets. Moreover, such refusal was less likely for assets such as mortgages, vehicles, and possessions, and more likely for items such as cash savings, stocks, and bonds. There were also associations, albeit small, between net worth and the likelihood of refusing to answer or not knowing the answer to a question. In contrast to this slight reluctance to report on their assets, those who possessed more assets were more likely to participate every year than those with fewer. Thus, although there is clear evidence for some response (and retention) biases, high retention rates combined with high response rates meant that little imputation of values was needed (Zagorsky 1999).

It is also important to mention those measures of income we did not include in our analyses. Most notably, the NLSY79 incorporates a constructed variable of “household income,” which combines the income of all respondents related by blood or marriage residing in the household (i.e., income from siblings, parents, and/or children living in the household, as well as spousal income). It does not, however, include partner income in the case of unmarried couples, even when the partner resides in the same household: the criterion for inclusion in the household measure is that people be related by either “blood or marriage.” For our purposes, this is problematic because partner income is likely to be more relevant to understanding childbearing decisions than the income of other relatives in the household. Although there are separate variables for partner and spousal incomes, they are not measured consistently, particularly for partners. Moreover, respondents were less certain about their spouse’s income than about their own income, and even much less certain about their non-spousal partner’s income. Given this, we focused only on the income and net worth of the main respondent and did not include information on partners or spouses. This is, of course, a clear limitation, because childbearing decisions are likely to be dependent on the income and net worth of both partners in a relationship.

### Ethnic Differences in Income and Net Worth

**Fig. 3:**
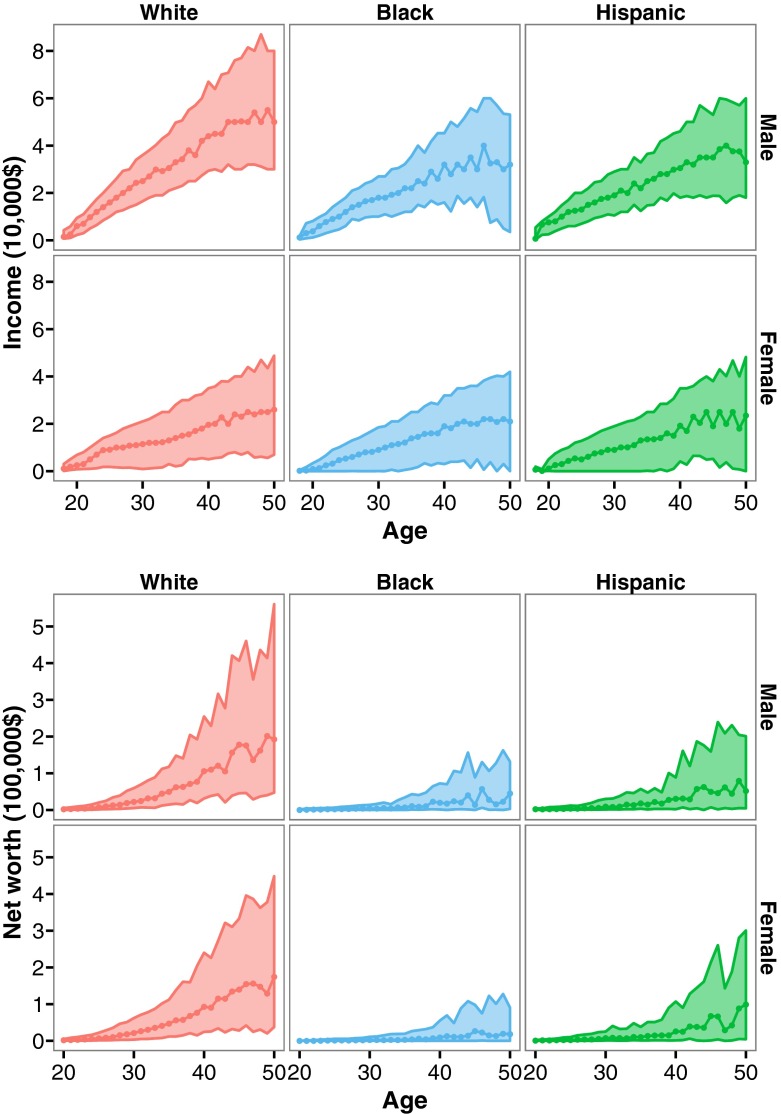
Income and net worth (in US dollars) across the life span for white, black, and Hispanic men and women

Examining the patterns of income and net worth across the life span, we find stark differences across ethnicities (Fig. 3). These replicate earlier studies from both the NLSY79 and other US samples (Emmons and Noeth 2015; Keister 2000). White respondents of both sexes acquire much greater net worth than black and Hispanic respondents, although there is substantial variation within whites. These ethnic differences in net worth are argued to result from differences in inheritance patterns (Keister 2000), education (Emmons and Noeth 2015), financial decision-making (Emmons and Noeth 2015; Keister 2000), discrimination, cumulative disadvantage, and early learning experiences (Emmons and Noeth 2015). White men also have much higher income compared with the other ethnic groups. For women, differences in income are much less pronounced across ethnicities, and women in general earn much less than their male counterparts.

These findings are important to consider in light of our research question. First, increases in income and net worth differ by sex and ethnicity in complex ways. For example, we might expect net worth to play a larger role than income in white women’s reproductive decision-making because (*a*) there is large variation in net worth and (*b*) average levels of net worth are much higher in white women than in women of other ethnicities, whereas this is much less pronounced in the case of income. Second, although we should be cautious of committing the ecological fallacy (Pollet et al. 2015), when we examine these aggregate patterns, it is clear that the groups with the highest income and net worth (white men and women) also report the lowest number of children (see Figs. 1–3). This suggests that other factors exert an influence on fertility besides wealth, or at least “material wealth,” which again implies that different ethnic groups may follow different reproductive strategies.

### The Cross-Sectional Analysis of the Association between Wealth and Lifetime Reproductive Success

Following this descriptive assessment of the available data, we now turn to our cross-sectional analysis of wealth in relation to LRS: a question that can only be dealt with effectively using cross-sectional data, given the nature of the dependent variable. Another reason for performing such an analysis is because it permits a direct comparison with another recent analysis of the wealth-fertility relationship in the NLSY79. Thus, we can provide a neat illustration of how differences in sample selection, along with variable selection and construction, can affect the outcomes of analyses.

Specifically, Hopcroft (2014) presented a cross-sectional analysis of NLSY79 data in which she showed that income in 2010 (when respondents were between 45 and 53 years old) was positively associated with the number of children ever born in men, but negatively associated in women. Here, we extend these analyses and offer our own cross-sectional analyses of the same data, in which we consider income across the life span. This is because, although the number of surviving children is appropriately measured at the end of the reproductive life span, the use of a single income measure taken at the same point in time is less appropriate. That is, income in 2010 was used as a predictor of the number of children born many years earlier, when it can have no causal influence on the decision to bear children. The use of such a measure also makes the implicit assumption that income in early life is strongly correlated to income in later life, which is not the case in these data (see ESM Figures 1–2). Given that income and net worth are measured in many more rounds prior to 2010, the use of only a single measure to represent these variables means a large amount of available information goes to waste. Finally, selecting one particular year allows the researcher unwarranted “degrees of freedom” in the choice of the year selected. As a result, we decided to investigate the association between wealth across the life span and lifetime reproductive success in order to provide a more comprehensive analysis. That is, rather than selecting a single year’s income and net worth as representative of an individual’s material wealth, we constructed income and net worth measures for each wave of data collection. Thus, our analysis represents a series of cross-sectional “snapshots” taken across the life span, as we explain below. Moreover, we simultaneously assess the effect of income and net worth by including both variables in all statistical models. This, in turn, required us to address two additional analytical issues: (1) the use of income and net worth in our statistical analysis and (2) selection of, and controlling for, confounding variables.

### Constructing the Income Variable

The top-coding of income (see above) along with the typical skew of income distributions (see Fig. 3), economic inflation over time, and the increase of income with age means that the income variable required transformation in order to generate consistency across years and ages before it could be used as a predictor. To account for these effects, and to ensure we could include the top-coded 2% in our sample, we converted this variable into quintiles within ages. A value of 1 for income, for instance, means that this individual was in the lowest 20% of his or her income group relative to individuals of the same age, whereas a value 5 indicated that the individual was in the highest 20% income group. We performed this standardization separately for the sexes because of the large difference in income between men and women. We performed a similar standardization for net worth. Quintile measures were included in our statistical models as continuous variables. Quintiles were used because a larger number of categories (e.g., deciles) was not feasible because too few cases were available or there was too little variation in income or net worth to enable them to be grouped into more categories; see Grundy and Read (2015) for similar analytical strategies. Hart (2015) also used quintiles but included them as a categorical variable in his analyses. In our case, sample sizes were too low to follow this strategy.

### Choice and Justification of Confounding Factors

In order to assess the effect of income and net worth on LRS, it is necessary to control for other variables that affect either the independent or dependent variable(s). Here, we included the following factors: country of birth (US or other), religion, whether the respondent lived in a rural or urban environment at age 14, region of the United States where the interview was held (Northeast; North Central; South; West), the number of siblings in 1979, maternal education, and respondent education. For reasons discussed above, we analyzed different sexes and ethnicities separately.

Religious differences are well-known to influence fertility (e.g., McQuillan 2004), as are geographical differences (reflected in the rural-urban designation, and location in the US). Country of birth has also been shown to be particularly important in explaining variation in fertility within Hispanics (Sweeney and Raley 2014). We included maternal education as a proxy for respondents’ socioeconomic background during childhood, which is thought to be predictive of fertility. Paternal education arguably would be a better proxy, but 14.7% of values on paternal education were missing, whereas only 5.5% were missing for maternal education. Thus, using paternal education would not only reduce sample size, but also bias the sample toward “nuclear” families. One’s own education is also a strong determinant of fertility (Skirbekk 2008), particularly in women, as well as being associated with income and net worth (e.g., Boshara et al. 2015).

Number of siblings is often positively associated with fertility, which can occur through a number of pathways: (*a*) having multiple brother and sisters may be indicative of high fecundity, and there is evidence for heritable variation in fertility that would support this inference (Kohler et al. 1999; Tropf et al. 2015a, b); (*b*) having multiple siblings may shape fertility intentions (whether positively or negatively). Our selection of control variables is thus substantially different from that of Hopcroft (2014), who included only sex, education, and “intelligence.” It is important to emphasize here that neither set of decisions is inherently correct, and that analyzing the data in a variety of ways furthers our understanding of the relationships that exist, and the extent to which they are robust. At the same time, it is also true that failing to account for highly influential factors such as sex and ethnicity may result in biased estimates.

### The Association between Income and Net Worth and the Number of Surviving Children

**Fig. 4:**
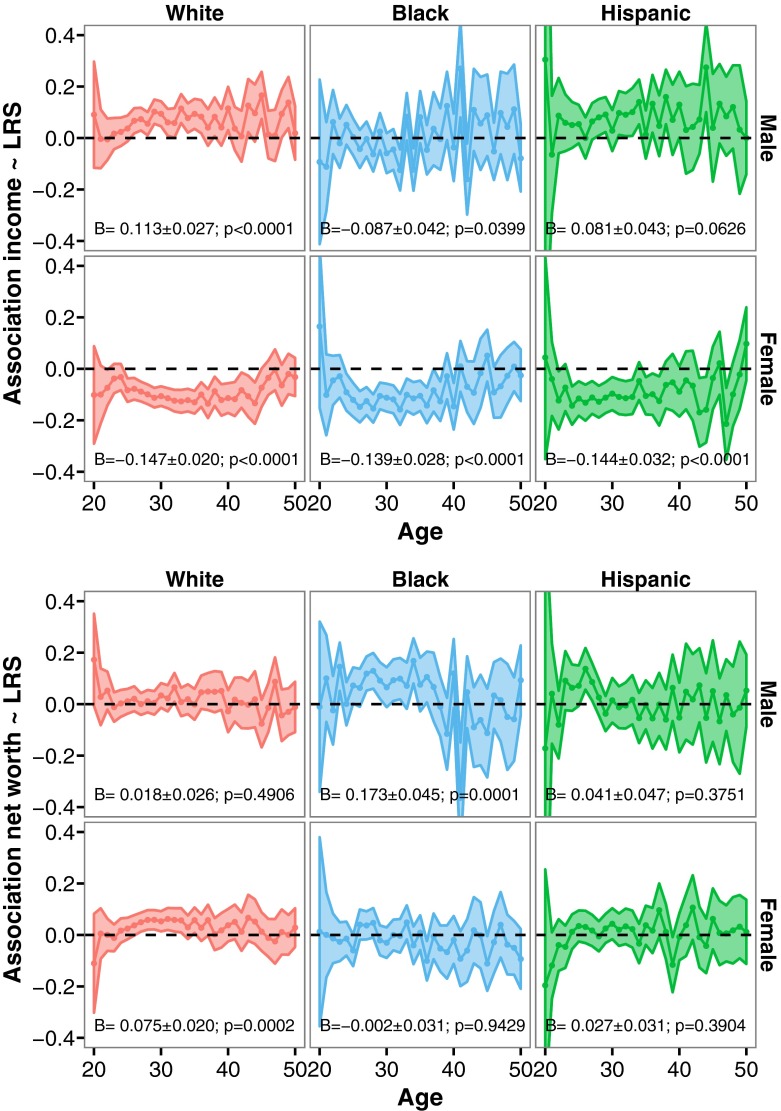
Poisson regression estimates (*B*; dot) and 95% confidence interval (*shaded area*) for the effect of income and net worth at a given age and lifetime reproductive success (LRS; note that LRS was only determined when the last age at interview exceeded the age of 44) for white, black, and Hispanic men and women. The Poisson estimate (*B*; plus standard error and *p* value) of the effect of an individual’s median income or net worth throughout life on LRS is presented in text at the bottom of each panel. With respect to effect size: e^B^ represents relative risk, with the interpretation that with one quintile increase, LRS would be increased by e^B^

To examine how measures of wealth at each age were associated with the number of surviving children in later life, we used data from the respondents across multiple waves of data collection. We thus examined how wealth from all individuals who reported their income at a given age was associated with the number of surviving children produced over their entire reproductive life span (see ESM for further details). Figure 4 shows the effects of income and net worth across the life span on lifetime reproductive success (for respondents who are aged 45 or older) from our Poisson regression analysis. So, for example, the effect seen at age 20 indicates the strength and direction of the effect of income earned when 20 years old on the number of surviving children at older ages. In women, across all ethnicities, a clear picture emerges: income is negatively associated with LRS across the life span, although the effects tend to become slightly less negative at older ages. For net worth, there is a consistent positive affect on LRS for white women only, between the ages of around 25 to 35.

In white and Hispanic men, the effect of income on LRS is almost always positive. For black men, in contrast, there is no consistent pattern. When it comes to net worth, we observe almost the opposite effect: for white and Hispanic men, net worth does not seem to be associated with LRS, whereas for black men, there is an apparent positive association between net worth and LRS, particularly between the ages of 25 and 35.

In addition to providing these snapshots of wealth at different ages, we also tried to create measures that captured overall income and net worth generated across an individual’s life. We calculated a median income across the life span for those respondents aged 45 and over who had reported on their incomes at least five times. We rounded this variable and again generated five wealth categories. We then did the same for net worth.

We found very similar results when using these measures (see Fig. 4 for model estimates). Across all women, median income across the life span was consistently negatively related to LRS. Furthermore, in white women, the median value of net worth was positively associated with LRS. Median income was positively associated with LRS in both white and Hispanic men, and there was a negative association for black men. In contrast, in black men, a positive association between net worth and LRS was observed (see ESM Figure 3 for correlations between income and net worth, that were positive but not particularly high). These results are very similar when analyses are conducted without controlling for confounding factors—if anything, the associations between income and LRS are slightly larger in magnitude (see ESM Figure 4).

### Cross-Sectional Analyses in Context

Overall, these results are in line with other studies which also show that, generally speaking, income is positively associated with LRS in men, and negatively in women (e.g., Barthold et al. 2012; Fieder and Huber 2007, 2012; Hopcroft 2006, 2014; Nettle and Pollet 2008; Weeden et al. 2006). However, our analyses extend this earlier work in several ways. First, we show that the effect of income differs across ethnicities, suggesting that studies that fail to include or control for ethnicity may generate a slightly misleading picture. Moreover, we find that net worth is positively associated with LRS among women and black men, suggesting that, for some groups, the stock of resources exerts a greater influence on reproductive decisions than the flow. We also observe that the effects of income and net worth are typically most pronounced during peak childbearing ages (i.e., between 25 and 35), suggesting that resources are particularly important at those ages. This indicates that using measures of income earned late in life could provide a biased account of the association between resources and LRS.

Although Fig. 4 plots the associations between our measures of wealth and LRS across the life span of our respondents, it consists nevertheless of multiple cross-sectional analyses. To gain a better understanding of the process of reproductive decision-making, and specifically how wealth might factor into the probability of producing offspring, longitudinal analyses are needed. We turn to these below.

## Longitudinal Analysis of the Association between Wealth and Reproductive Outcomes

It has long been recognized that a better understanding of reproductive decision-making can be gained by looking at reproduction as a series of decisions distributed across the life span, rather than treating LRS (or desired family size) as a one-shot decision made at a single point in life (Goldberg 1960; Namboodiri 1972, 1975; Werding 2014). Although individuals may have an initial preference for children and a desired family size (e.g., a two-child norm), desires and preferences can change (Liefbroer 2009), and child-rearing experiences can and do shape subsequent fertility decisions (Margolis and Myrskylä 2015). Moreover, each birth may be influenced by a different set of factors—something that may very well be true for income and net worth. Treating reproduction as a process also fits with the life-history framework used by (human) behavioral ecologists (Alvergne and Lummaa 2014): current circumstances influence how future trade-offs will be resolved. There is also a statistical benefit to such an approach: the temporal ordering of the variables (with measures of income and net worth taken prior to, rather than following, a birth or even all births) allows for an assessment of associations that are less prone to reverse causality (something that could explain the negative association between female income and LRS—in other words, women with more children have lower income because they earn less after having children [see Stulp and Barrett 2016a for discussion]). Finally, longitudinal analysis can account for censoring (in this instance, the inclusion of individuals who have not yet completed their reproductive life span) in the data.

### Analytical Strategies and Nonindependence

We used discrete-time event history models (Mills 2011; Steele 2011; logistic mixed models) to assess the effect of wealth on the probability of birth, which meant that we transformed our data into person-years, such that each individual had a line of data for each year from the age they entered the dataset (in 1979) and were minimally 18 years old until the age they were last interviewed. For simplicity, we refer to this effect on fertility as “probability of birth,” but it should be borne in mind that this refers to the probability of a birth within a discrete time period following the income measure, and not the overall probability of birth. We modeled only the first three births because (1) there were too few births at higher parities for this type of analysis and (2) the ages of the first three births are directly available and have been checked for consistency and accuracy by the data developers. Participants were censored at the year of last interview, at the age of 45, or after the birth of a third child. We included a random intercept for the respondent to account for the fact that multiple births are nested within the same individual. Modeling the first three births simultaneously is of utmost importance; Kravdal (2001), for instance, showed that, although education was positively associated with the transition to a second and third birth when modeled separately, the association became negative when modeling all births simultaneously (this is due to problems of endogeneity—a particular biased subsample of women is left for analysis when only second and third births are focused on).

**Fig. 5:**
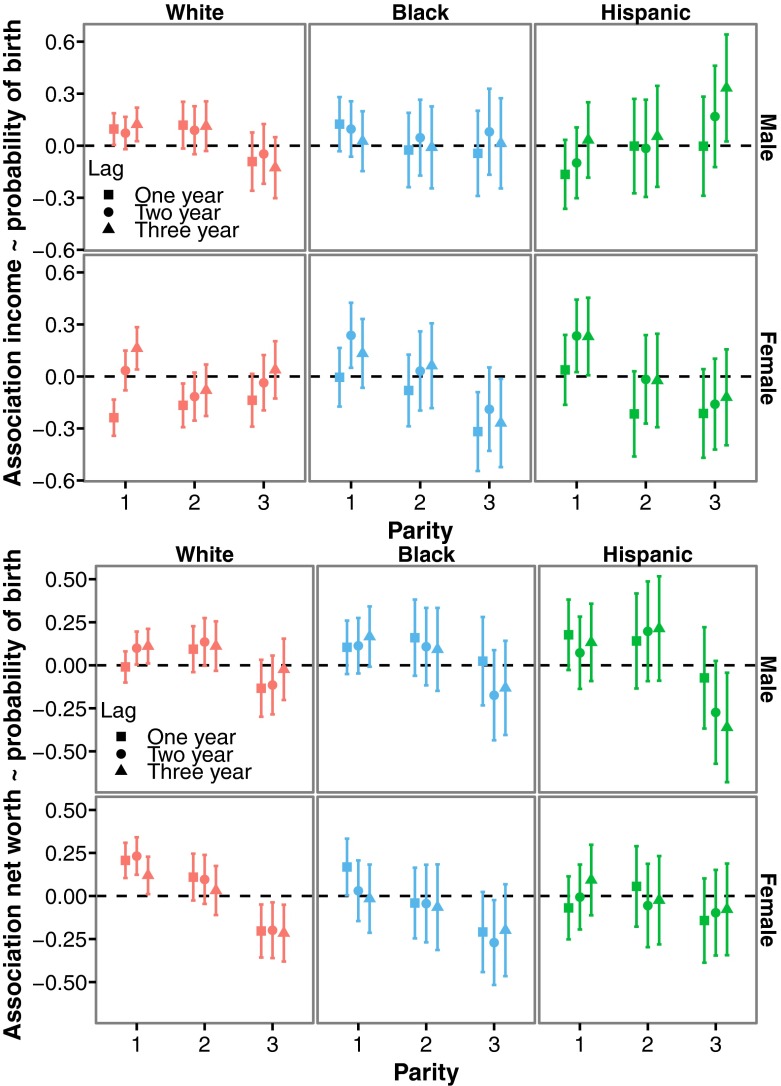
Logistic mixed model parameter estimates (*B* and 95% confidence interval) of income and net worth measured one, two, or three years before the time of interview (Lag “One year,” “Two year,” and “Three year,” respectively) on the probability of a first, second, or third birth (Parity 1, 2, and 3, respectively) within a time period of a year for white, black, and Hispanic men and women. Interactions between parity and income, and parity and net worth were always included, independent of *p*-value. Individual was included as a random intercept. With respect to effect size: e^B^ represents odds-ratio, with the interpretation that for a randomly chosen individual, the odds of having a birth with a value of wealth of X are e^B^ times the odds when having a wealth of X−1

We always included time-varying measures of income and net worth simultaneously, and we also included interactions with parity to see whether the effect of income and net worth on probability of birth was different for each parity progression. That is, we could ask whether these constituted different decisions with different underlying motivations (Namboodiri 1972; Philipov et al. 2006). Moreover, income and net worth measures were lagged by one, two, and three years, and these lagged variables were analyzed separately (note that the lag is from the time of interview and not from the time of birth). See Fig. 5 for an overview of the results. Also note that we are more interested in the overall patterns of income and net worth across sex and ethnicity, and less so in individual significant results. Results are very similar when confounding variables are not included in our analyses (although relationship status seemed important; see ESM Figure 5).

### Longitudinal Patterns between Wealth and Fertility: Investigating Decision-Making

Among white men, lagged income positively predicted the probability of first and second births but had a negative influence on the probability of a third birth. Income in the previous year was typically the strongest predictor. With respect to net worth, lagged values from the previous two and three years positively predicted the probability of a first and second birth in white men (independently of income). Again, there was a negative influence on the probability of a third birth, although the magnitudes of the effects are low. For black men, we found no evidence for an association between income and births, although, in contrast to the cross-sectional negative association, income in previous years seemed to positively rather than negatively predict the probability of births. A similar picture emerges for net worth. In Hispanic men (where sample sizes were low), we found no evidence for an effect of our wealth measures, although income lagged by one and two years was negatively associated with the probability of parenthood, whereas income lagged by two and three years was positively associated with the probability of a third child. For net worth, the opposite pattern emerged: when the effect of income was positive, the effect of net worth was negative.

Among white women, income in the previous year was negatively associated with the birth of a first, second, and third child, whereas income two and three years previously was typically not associated with the probability of a birth. One exception is the positive effect of income received three years prior to having a first birth. In stark contrast, net worth in the previous three years positively predicted the probability of a first and second birth and negatively predicted the probability of a third birth. For black women, we found that income lagged by two (and to a lesser extent three) years positively predicted the probability of becoming a mother, as well as net worth in the previous year. In contrast, income was not associated with the probability of a second child, and it was associated negatively with the probability of a third child. Net worth also appeared to negatively predict the probability of a third child. Finally, among Hispanic women, income from the previous two and three years positively predicted becoming a mother, whereas income seemed to be negatively related to the probability of having a third child. Net worth did not seem to have any effect on the probability of a birth among Hispanic women.

In conclusion, income and net worth affect childbearing decisions differently: differences were not only observed across the sexes, but also across parities and ethnicities. Income in the previous year was typically negatively associated with the probability of birth in women. One possibility is that this reflects the influence of maternity leave (both in the period preceding the birth as well as after; at the time of interview, respondents were asked about their income across the previous year—a pregnancy/birth could coincide with that period). Unpaid maternity leave is typical for the United States and would reduce yearly income during the early postnatal period. In addition, although maternity leave does not begin until shortly before a birth, a loss of income during pregnancy may also occur if women decrease their working hours in anticipation of the upcoming birth. It is interesting to see that, for white women only, income received two or three years earlier was not associated with the probability of birth. Combined with the previous cross-sectional patterns, which indicated that median income throughout life was negatively associated with lifetime reproductive success, this suggests that having children actively impedes the generation of income by such women, rather than high income simply reducing the probability of births at a later stage. Support for this also comes from the observation that net worth is positively associated with becoming a mother and having a second child. In other words, it would be wrong to conclude that women are not translating their resources into having children. Among black and Hispanic women, income received two or three years previously positively predicts the probability of becoming a mother, whereas for white women only income received three years previously did. This suggests that women may postpone the decision to become a parent until they have sufficient income, or have a job of a particular standing (see Scott and Stanfors 2011 for a similar case in Sweden).

For men, the effects of income were most pronounced among whites, where it positively predicted the probability of both becoming a father and having a second child. Net worth was also positively associated with a first and second child, this time across all ethnic groups, although support for these associations was rather weak (except perhaps for white men).

Net worth was almost always negatively associated with the probability of a third child (across both sex and ethnicity), a pattern that was particularly clear among both white and black women. For white men, there were also contrasting effects of income and net worth on the probability of different births: The relationship was positive with respect to the first two births, but negative with respect to the third birth. This suggests that, when it comes to resources, the transition to a third child reflects a somewhat different decision-making process (see also above).

### Reflections on the Association between Wealth and Fertility

Our analyses, both cross-sectional and longitudinal, revealed a complex picture of how resource availability influences fertility and transitions to parenthood and higher parities. Income and net worth had separate, and sometimes contrasting, effects on lifetime reproductive success and fertility transitions. Cross-sectional patterns revealed that income and net worth measured during the period in which most births occur (between 25 and 35 years of age) were more strongly associated with lifetime reproductive success than wealth measures from later in life, suggesting that people do factor wealth into their fertility decisions during their childbearing years.

For women, median income across the life span was consistently associated with lower lifetime reproductive success—a pattern often observed. There are, however, two lines of evidence to suggest that this is not because richer women fail to translate their resources into more births, but because women receive less income when children are born: (1) net worth is positively associated with fertility (at least in white women) and (2) income received in the year prior to the birth shows the strongest negative association with the probability of a birth, whereas this effect is not observed for income for two or three years prior to a birth (indeed, this income measure sometimes had a positive effect, at least on becoming a parent). This reverse causality, where women pay a substantial monetary cost for giving birth (sometimes referred to as the “motherhood wage gap”), is pronounced in the United States, where paid maternity leave is rare and considered a luxury (Scott and Stanfors 2011). In Sweden, for instance, maternity and paternity leave are generous, and the amount of governmental pay mothers receive during maternity leave is strongly determined by their income; it is not surprising, therefore, to see that income positively predicts motherhood in this population (Dribe and Stanfors 2010; Scott and Stanfors 2011; Stanfors 2014). This indicates that fertility decisions should be considered within the institutional context of the particular population under study, and that we shouldn’t regard industrial societies as homogenous. Clearly, the United States is not representative of all industrial populations, and our results are unlikely to generalize across all high-income nations (see also Stulp and Barrett 2016a; Stulp et al. 2016). This further suggests that general economic theories of fertility, which are heavily based on the US system, are unlikely to be easily translated to other populations without considerable revision (see also Balbo et al. 2013).

Our analyses also show the value of considering both cross-sectional and longitudinal patterns. In particular, our longitudinal analyses suggest an alternative interpretation of the negative cross-sectional relationship between female earnings and fertility outcomes (see also Hart 2015). The variable nature of our wealth measures, in addition to the possible negative causal effects of children on income and net worth, would argue against simplistic cross-sectional studies. Having said this, our cross-sectional analyses allowed us to investigate overall LRS in relation to wealth, so perhaps it is more reasonable to suggest that cross-sectional measures complement longitudinal analyses.

While it is true that our analyses of the association between wealth and fertility go beyond those typically published in the evolutionary literature, it is also true that our analyses have only scratched the surface: clearly there are many other possible avenues to explore that will provide a richer understanding of the patterns at hand. For example, we could investigate the extent to which the positive effects of wealth on fertility in men are driven by partner choice (Barthold et al. 2012; see also ESM Figure 5). We could also test whether fluctuations in wealth (or unemployment) are more important in explaining fertility decisions than absolute wealth (Currie and Schwandt 2014; Modena et al. 2014). In addition, we could also consider the extent to which differences in fertility preferences explain our results, whether resources allow individuals to (more easily) achieve their fertility goals, and whether resources actively shape fertility preferences (Modena et al. 2014). A closer examination of the effects of the confounding variables would similarly pay dividends. It is here that the value of large secondary databases becomes apparent, for the NLSY79 dataset allows for the testing of all these questions.

### Some Suggestions about Reproductive-Decision-Making Mechanisms

Given that we have only scratched the surface with the present analyses, our ability to point to specific fertility-mechanisms is inevitably rather limited. Nonetheless, our analyses, combined with previous literature, do indicate certain decision-making processes, and the results also give pointers where to look further.

It may seem to be a rather obvious point, but the fact that a large majority of people produced at least one child, in an environment where fertility can be quite tightly and consciously controlled, suggests that many people wish to become parents: that is, children do not seem to be merely a(n undesired) side-effect of searching for opportunities to engage in sex. The active desire for children may explain why people persist in starting a family in the face of quite extreme economic costs (Morgan and King 2001; Rotkirch 2007). Indeed, from an economic perspective, it is often difficult to understand why people have any children at all (Coleman 2000), and an understanding of evolutionary processes may well pay dividends here (see also Sear 2015). Of course, the possibility remains that people in certain socioeconomic strata may be less able to control their fertility as well as they might wish, resulting in unintended births (e.g., Musick et al. 2009): certain sectors of society may find it difficult to access the contraceptive devices needed to control fertility, or to access termination services in the case of unwanted pregnancy, given variation in access to family planning services in the United States.

The distributions shown in Fig. 1 seemingly point to the existence of additional mechanisms. The fact is that the number of people with either no children at all or an only child was much lower than expected, whereas a family size of two children was much more frequent than expected by chance. This suggests that US families prefer to have two children and are not inclined to have only one child. This, then, requires an explanation of why this should be the case, and the decision-making process that underlies such reproductive choices (or indeed the lack of them). Both behavioral ecological as well as cultural evolutionary ideas have been put forward to explain patterns of this kind. For example, one reason people apparently avoid having only one child (Blake 1974; Morita et al. 2015) is argued to be because parents believe that an only child will not be properly socialized without siblings (Blake 1974). Such an explanation cannot, however, account for why so many people produce only two children and not have even more (after all, more siblings means more socialization: Lawson and Mace 2010a). Behavioral ecological and economic explanations stress the importance of costs of raising children to explain this phenomenon: a third child may come at a substantially higher cost than a second child. With increasing family sizes, there are significant costs to offspring quality, measured in terms of health and well-being, and levels of education and wealth accrued in life (Keister 2003; Lawson and Mace 2011). Thus, one could interpret our finding showing that net worth positively predicts the probability of a first and second child, but is unrelated or even negatively related to the probability of third, as evidence that the decision to have a third child is indeed different (see also Namboodiri 1972; Philipov et al. 2006), and that they are perhaps more costly (and perhaps particularly so for wealthy people; Lawson and Mace 2010b).

An alternative explanation is that resources play a role in initially becoming a parent as well as permitting the production of a second (“obligatory”) child, but no longer exert an influence (or at least not a strong one) on the production of a third child. It seems possible that the transition to parities above two is much less dependent on resources and that other influences, such as decisions made by those in an individual’s social network or other idiosyncratic factors gain prominence (e.g., Angrist and Evans 1998; Balbo and Barban 2014). In other words, economic factors may influence the desired goal of two children (Carey and Lopreato 1995; Morita et al. 2012; Sobotka and Beaujouan 2014), whereas the desire for higher parities may be more strongly influenced by noneconomic factors. As an example, families are more likely to have a third child when the first two children are of the same sex (Angrist and Evans 1998). The decision to have more than two children in this case seems to satisfy the desire of parents to at least have one child of each sex—something that is potentially difficult to reconcile in a standard life-history approach. Having said this, if the sexes pay different economic, social, and (potentially) fitness dividends, it may be possible to situate such findings within behavioral ecological theorizing. In either case, cultural evolutionary ideas may also be informative for explaining such patterns (see Colleran 2016; Stulp and Barrett 2016a for further discussion).

A further indication of how non-resource-based influences may be important comes from the substantial variability that is observed in reproductive strategies in our sample. It is clear that white, black, and Hispanic men and women have different fertility schedules, and it is well known that childbearing occurs in different contexts (Hartnett 2014; Sweeney and Raley 2014): for example, differences in relational context, whether births are planned, patterns of sexual activity, contraceptive use (see Musick et al. 2009 for a similar point with respect to educational strata). This is also apparent when examining the different relationships between measures of wealth and fertility (for example, in black men income is negatively, and net worth is positively, related to LRS, whereas for white and Hispanic men, only income is positively associated with LRS). Furthermore, the finding that white men and women have much higher incomes and net worth than other ethnicities, yet have the lowest fertility, suggests that economic resources are not the only factor influencing reproductive decisions. For example, ethnic differences in the composition of networks may provide a context in which higher childbearing can be supported despite limited resources (Bereczkei 1998; Chan and Ermisch 2015; Geronimus 1996; Turke 1989). Such ethnic differences may also arise from differences in perceived costs of raising children, or from different values and ideas considering having (many) children (Hartnett 2014). Suffice it to say, variation in reproductive strategies observed across different ethnicities suggest that different decision-making mechanisms are at play.

## Conclusion

Clearly, the factors influencing fertility decisions in industrial populations represent a fascinating, but highly complex, issue. The possible evolved desires highlighted by evolutionary psychologists, the life-history trade-offs as studied by human behavioral ecologists, and the norms and social learning strategies studies by gene-culture coevolutionary theorists are all needed to explain this behavior. Our analysis of the relationship between wealth and fertility confirms that the evolutionary story is anything but simple. Although it is clear that arguments against measuring fertility in industrial populations are unfounded, it is also true that we need a much deeper understanding of the mechanisms at play in order to understand low fertility in contemporary populations. Our main point, however, is that the analyses of large-scale databases offers a fruitful means of enquiry into evolutionary questions, as long as the limitations and challenges of such databases are recognized, and care is taken in the selection of relevant samples, the control of confounding variables, and the construction of an appropriate statistical modeling strategy.

## Electronic supplementary material

ESM 1 (PDF 306 kb)

## Footnotes

1 Although our use of the term “fertility” conforms to the definition used in the social sciences (i.e., the number of children born) rather than the biological definition, which considers fertility to be the ability to conceive, we extend beyond this strict definition to encompass all aspects of reproductive behavior in our discussion of why measuring fertility is important.
