# Supplementary material for: The Reproductive Ecology of Industrial Societies, Part II: The Association between Wealth and Fertility
Source: Hum Nat. 2016 Sep 26;27(4):445–70. doi: 10.1007/s12110-016-9272-9 (PMC5107208; doi:10.1007/s12110-016-9272-9)
Supplement: Supplementary file 1 — (PDF 306 kb) [file 12110_2016_9272_MOESM1_ESM.pdf]

Electronic Supplementary Material (ESM)

The Reproductive Ecology of Industrial Societies, Part II: On the Association between Wealth and Fertility

Human Nature 27(4), 2016, DOI 10.1007/s12110-016-9272-9

Gert Stulp[a,b*], Rebecca Sear[a], Susan B. Schaffnit[a], Melinda C. Mills[c] & Louise Barrett[d]

[a] Department of Population Health, London School of Hygiene & Tropical Medicine, Keppel Street, London WC1E 7HT, UK

[b] Department of Sociology, University of Groningen / Inter-university Center for Social Science Theory and Methodology (ICS), Grote Rozenstraat 31, 9712 TG Groningen, The Netherlands

[c] Department of Sociology and Nuffield College, University of Oxford, Manor Road, Oxford OX1 3UQ, UK

[d] Department of Psychology, University of Lethbridge, Alberta T1K 3M4, Canada

* Corresponding author: g.stulp@rug.nl

Contents

Methods 2
- Variables 2
- Analysis 2
- Imputation 4

References 4

Table ESM 1. Descriptive statistics of men who remained in the sample until at least the age of 45 5
Table ESM 2. Descriptive statistics of women who remained in the sample until at least the age of 45 6

Figure ESM 1. Spearman rank correlations between income across different survey waves 7
Figure ESM 2. Spearman rank correlations between net worth across different survey waves 7
Figure ESM 3. Spearman rank correlations between income and net worth across different survey waves 8
Figure ESM 4. Poisson regression estimates for the effect of income and net worth at a given age 9
Figure ESM 5. Logistic mixed model parameter estimates of income and net worth 10
Figure ESM 6. Logistic mixed model parameter estimates of income and net worth 11

Methods

The response rate for those believed to be alive in 2012 is 78.7%. Elaborate and well-organised information, including that on sampling procedure, interview details, and confidentiality and ethics can be found at: http://nlsinfo.org/content/cohorts/nlsy79/.

Variables

Variables that were constructed by the Center for Human Resource Research had undergone consistency and quality checks, so we opted for these constructed variables whenever possible. Moreover, for respondent background information, we used the first wave of data collection, when respondents answered most of the questions. From this wave, we included sex, country of birth (US or other), ethnicity, religion that they were raised in, whether they lived in a rural or urban environment at age 14 ("town or city", "country", "farm or ranch"; we collapsed the latter two), and region of the US at time of the interview (Northeast; North Central; South; West; note that more detailed information on geographical area is not available in the publicly available data), the number of siblings 1979, and maternal education (years of schooling).

We categorized ethnicity into white, black, and Hispanic. Other ethnicities (i.e., Native American, Asian, or Jewish, and all other) were too few in number, and were dropped from analyses. When the respondent mentioned only one Ethnic origin, we used this information, and we used the ethnicity with which the respondent identified most closely in cases where they reported multiple ethnicities. With respect to religion, many possible denominations were recorded, which we summarized into four categories (based on religious classification and frequencies): Protestant, Roman Catholic, Other, and No Religion. As recommended and when available, we used the birth year as recorded in 1982; in the remaining cases, we used the value given in 1979. With respect to the education of the respondent: at every wave, the highest educational grade completed was obtained, ranging from none to more than 8 years of college. We created a variable "highest educational grade completed". On the basis of the frequency distribution and the US educational system, we classified education into a categorical variable with 5 options: "Not finished high-school" [<12th grade], "High-school education" [12th grade], "Some college education" [1st – 3rd year of college], "Bsc and higher" [4th year college– 8th year college or more]. We used a similar classification for maternal education. Financial support was available to check the consistency and quality of the fertility history data from 1982 onwards, and the constructed variables are therefore highly recommended. We used the information from the constructed variables of the ages at first, second, and third birth for our longitudinal analysis, and we used information on the birth and death dates of the children, to construct the number of children ever born, the number of deceased children, and the number of surviving children. Whenever parents reported on the year of death of a child, the child was counted as deceased, irrespective of the age at death.

Analysis

For our cross-sectional analyses of income and net worth on lifetime reproductive success (i.e. number of surviving children), we used Poisson regressions, and we included the following control variables: the year of birth, ethnic and religious background, country of birth, urban or rural, region of the US, number of siblings, maternal education, respondent education, and the age at last interview. Note that for all cross-sectional analyses we only included individuals aged 45 and over. Also note that the variable country of birth was dropped for the analyses on black men and women, because there were too few cases (and sometimes no cases) of individuals born outside the US. For our longitudinal analyses, we used discrete-time event history models (Mills,

2011; Steele, 2011), which meant that we transformed our data in person-years, such that each individual had a line of data for each year from the age they entered the dataset (in 1979) until the age they were last interviewed. Each person-year contains data on fixed factors of the respondent (i.e., the year of birth, ethnic and religious background, country of birth, urban or rural, region of the US, number of siblings, maternal education, and respondent education), as well as time-varying variables, and importantly, whether an event of interest occurred in that year (e.g., whether a first, second, or third birth occurred). The time-varying variables that we included were: i) whether the respondent was currently in a relationship (either by mentioning a spouse, a partner, or 'other'); and ii) our wealth variables.

In essence, discrete-time event history models are logistic regression models that model the probability of an event per unit time (a year in this study), which can vary along the other factors in the model. Importantly, individuals that have not experienced an event, or that have been in the data for only a few years, can still be incorporated in the analyses (i.e. it includes censored cases). Using discrete-time event history models, we modelled the likelihood in a given year of a first, second, and third birth simultaneously (we did not include data on higher parities, because the probabilities of fourth births and higher parities were low for these type of analyses and less accurate information was available on the timing of birth at these higher parities). The data thus consisted of several person-years per individual; the first age included was age 18, because questions on income were only asked from age 18 onwards (this meant that the events before the age of 18 were excluded, although these individuals could still be in the analysis at higher parities). Participants were censored at the year of last interview, at the age of 45, or after the birth of a third child. In these models, time was measured in years since age 18 and was restarted after every birth (Steele, 2011). Both time (years since age 18/birth) as well as a squared term of this variable were included in all models to account for the fact that the probability of a birth is a non-linear function of age (or time since last birth). We modelled all births simultaneously; this was achieved by including a time-varying factor of 'Parity' that indicated whether a year was preceding a first, second, or third birth. Additionally, we included an interaction between our timing variable and parity, because the time until a birth occurred is likely to be much different for the first, second, and third birth. We included a random intercept for the respondent in these models to account for the fact that multiple births occur within one individual (sometimes referred to as frailty; Mills 2011). Income was always lagged in these models, such that the income received in the preceding year would predict a birth in a subsequent year. We always modelled income and net worth simultaneously. Moreover, we used three different versions of these different forms of wealth: income/net worth in the previous year of the interview date (a lag of one year), the year before that (a lag of two years), and the year before that (a lag of three years). Importantly, we always included both the interaction between income and parity, and net worth and parity, because we were specifically interested in whether wealth may have different effects on having a first, second, or third birth. Not all mixed models converged, which probably relates to the inclusion of a large number of control variables. When we set the number of adaptive Gauss-Hermite quadrature points to zero (which comes at a cost of accurately estimating the random effects), all models converged. Estimates from these models and the non-converging models are nearly identical (see ESM Figure 6). Estimates of the standard mixed models are presented in the main text. General linear models without random intercepts were also ran to validate the results, and these were also very similar (see ESM Figure 6).

Imputation
Since births do not occur very often, it is often the case that the year of birth of a child coincides with a year in which either no data was collected, or when a respondent did not respond. Such events would then be excluded from the statistical model. To prevent these events from being excluded in the analyses, we imputed our time-varying variables. For relationship status, we imputed the last known value. In contrast, for education, we imputed a linear approximation, meaning that values between time points will be imputed linearly along the years (see Zagorsky (1999) for similar strategy relating to wealth). For example, if education has a value of 8, then two years of missing values, and then a value of 9, this approximation will impute $8^{1/3}$ and $8^{2/3}$ for the years in between. We would subsequently round these variables, such that imputed variables could not deviate from original values, leading to a series of 8-8-9-9 in this case (with the middle 8 and 9 imputed).

These variables typically do not change much over time, and it is unlikely that the imputed values will have strong effects on our outcomes. Our variables of central interest, however, are those related to measures of wealth that may vary more over time. For these variables, we similarly used a linear approximation, however, we added some further restrictions to these imputations. If income had not been measured for five years in a row, we did not impute (note that we did not exclude these individuals, we just did not include those person-years for which income was missing). Importantly, our results were similar when we analysed only the non-imputed wealth variables.

All analyses were performed in R (R Development Core Team, 2008) including the use of the lme4 package. Graphics were produced using ggplot2 (Wickham, 2009).

References
Mills M. 2011. Introducing survival and event history analysis. London: Sage.
R Development Core Team. 2008. R: A Language and Environment for Statistical Computing.
Steele F. 2011. Multilevel Discrete-Time Event History Models With Applications To the Analysis of Recurrent Employment Transitions. Aust N Z J Stat 53:1–20.
Wickham H. 2009. ggplot2: Elegant Graphics for Data Analysis (Use R!). New York: Springer.
Zagorsky JL. 1999. Young baby boomers' wealth. Rev Income Wealth 45:135–156.

Table ESM 1. Descriptive statistics of men who remained in the sample until at least the age of 45.

Ethnicity	White Mean /%	SD / N	Black Mean /%	SD / N	Hispanic Mean /%	SD / N
**Age last interview**	51.25	2.49	51.38	2.40	51.21	2.61
**Was born in US?**		1152		536		376
Yes	98%	1134	98%	523	70%	264
No	2%	18	2%	13	30%	112
**Urban or rural?**		1150		531		375
Urban	75%	862	85%	452	88%	330
Rural	25%	288	15%	79	12%	45
**Region of US?**		1135		527		364
North East	21%	235	17%	91	18%	66
North Central	36%	405	21%	113	7%	25
South	28%	313	54%	283	24%	88
West	16%	182	8%	40	51%	185
**Religion**		1037		475		358
Protestant	55%	567	90%	426	4%	15
Rom. Cath.	38%	395	5%	24	94%	335
No religion	6%	59	5%	23	2%	7
Other	2%	16	0.4%	2	0.3%	1
**Education**		1153		536		376
No high school	8%	88	9%	46	22%	84
High school	42%	485	48%	257	45%	170
Some college	21%	240	24%	131	22%	82
Bsc or more	29%	340	19%	102	11%	40
**Education mother**		1109		495		344
No high school	25%	276	46%	227	77%	265
High school	53%	583	39%	191	17%	58
Some college	12%	134	9%	44	2%	7
Bsc or more	10%	116	7%	33	4%	14
**# Siblings in '79**	2.74	1.91	4.06	2.89	4.62	2.97
**#Children born**	1.73	1.32	2.02	1.67	2.28	1.6
**#Surviving children**	1.71	1.31	1.99	1.65	2.24	1.58
**Ever married**	89%	1010	74%	388	86%	315
**Ever had birth**	78%	775	79%	377	83%	258
**Ever had 2nd birth**	60%	598	58%	277	68%	212
**Ever had 3rd birth**	25%	244	34%	161	43%	136
**Age 1st marriage**	25.02	5.58	27.38	6.74	24.44	6.56
**Age 1st birth**	27.28	6.12	24.84	6.01	24.84	6.18
**Age 2nd birth**	30.23	5.81	28.06	6.15	27.90	6.02
**Age 3rd birth**	32.21	6.11	30.75	6.45	30.20	5.69

Table ESM 2. Descriptive statistics of women who remained in the sample until at least the age of 45.

Ethnicity	White Mean /%	SD / N	Black Mean /%	SD / N	Hispanic Mean /%	SD / N
**Age last interview**	51.40	2.51	51.46	2.41	51.65	2.57
**Was born in US?**		1248		810		469
Yes	97%	1211	98%	790	72%	340
No	3%	37	2%	20	28%	129
**Urban or rural?**		1246		804		467
Urban	76%	943	80%	644	89%	417
Rural	24%	303	20%	160	11%	50
**Region of US?**		1225		800		464
North East	20%	250	16%	130	22%	101
North Central	33%	399	18%	148	8%	38
South	30%	366	59%	475	28%	129
West	17%	210	6%	47	42%	196
**Religion**		1116		709		450
Protestant	58%	649	88%	624	7%	30
Rom. Cath.	36%	401	8%	60	92%	415
No religion	4%	50	4%	25	1%	4
Other	1%	16	0%	0	0.2%	1
**Education**		1248		810		469
No high school	6%	70	8%	61	19%	89
High school	40%	504	40%	323	36%	170
Some college	26%	319	33%	271	29%	135
Bsc or more	28%	355	19%	155	16%	75
**Education mother**		1208		744		435
No high school	29%	356	53%	398	76%	330
High school	49%	588	34%	255	18%	77
Some college	11%	134	8%	56	5%	22
Bsc or more	11%	130	5%	35	1%	6
**# Siblings in '79**	2.90	2.03	4.48	3.05	4.52	3.1
**#Children born**	1.86	1.23	2.2	1.53	2.46	1.59
**#Surviving children**	1.82	1.2	2.14	1.49	2.41	1.57
**Ever married**	93%	1147	73%	571	87%	396
**Ever had birth**	83%	920	85%	628	88%	374
**Ever had 2nd birth**	65%	727	69%	507	77%	326
**Ever had 3rd birth**	26%	292	41%	299	43%	184
**Age 1st marriage**	22.88	5.71	24.99	7.14	22.40	5.72
**Age 1st birth**	24.58	5.70	21.18	5.41	22.25	5.33
**Age 2nd birth**	27.45	5.34	24.62	5.65	25.58	5.46
**Age 3rd birth**	29.51	5.29	26.74	5.14	27.77	5.31

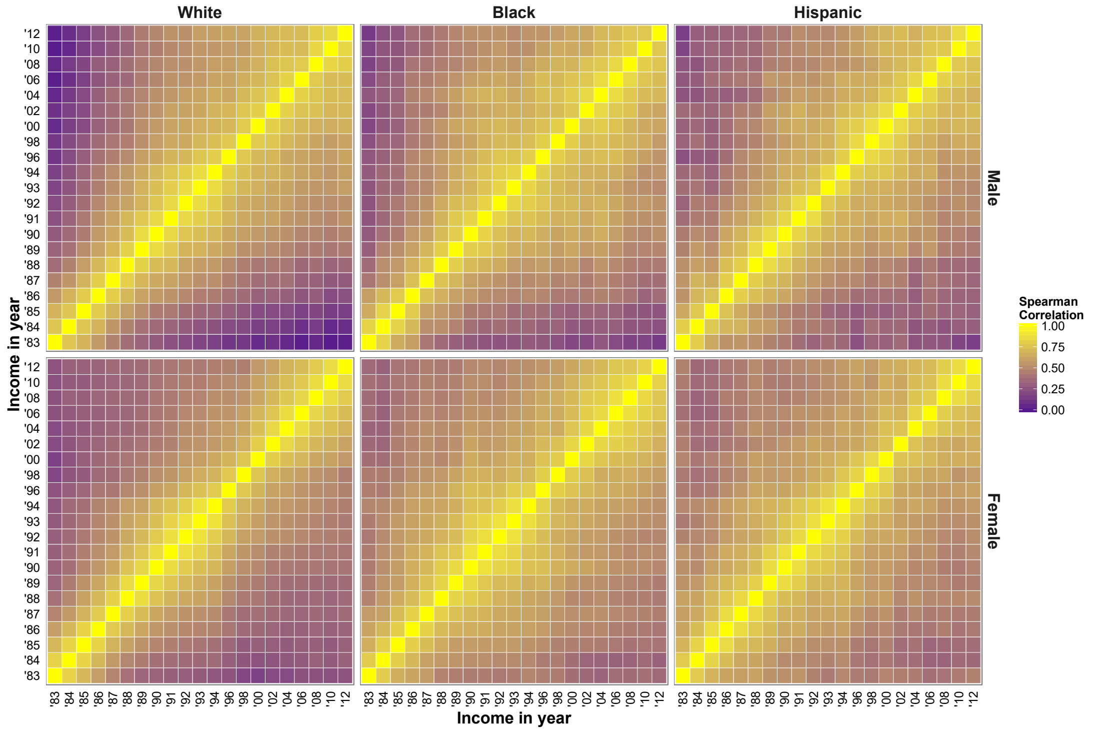

Figure ESM 1. Spearman rank correlations between income across different waves of the survey (minimum: 0.01; maximum: 0.88).

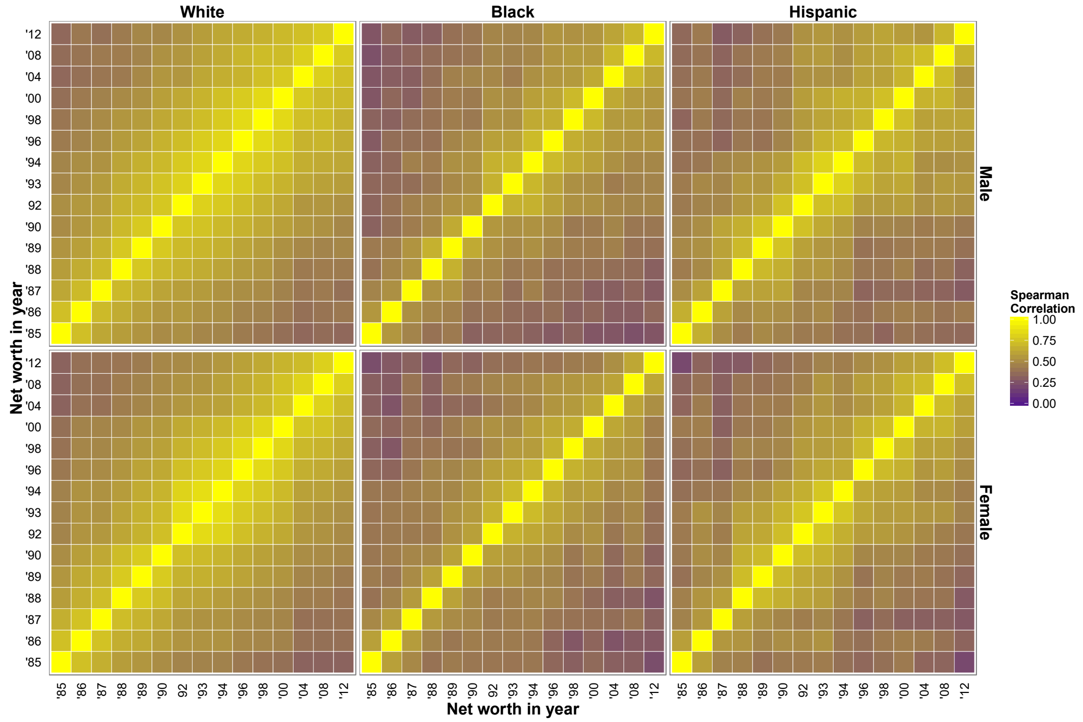

Figure ESM 2. Spearman rank correlations between net worth across different waves of the survey (minimum: 0.21; maximum: 0.86).

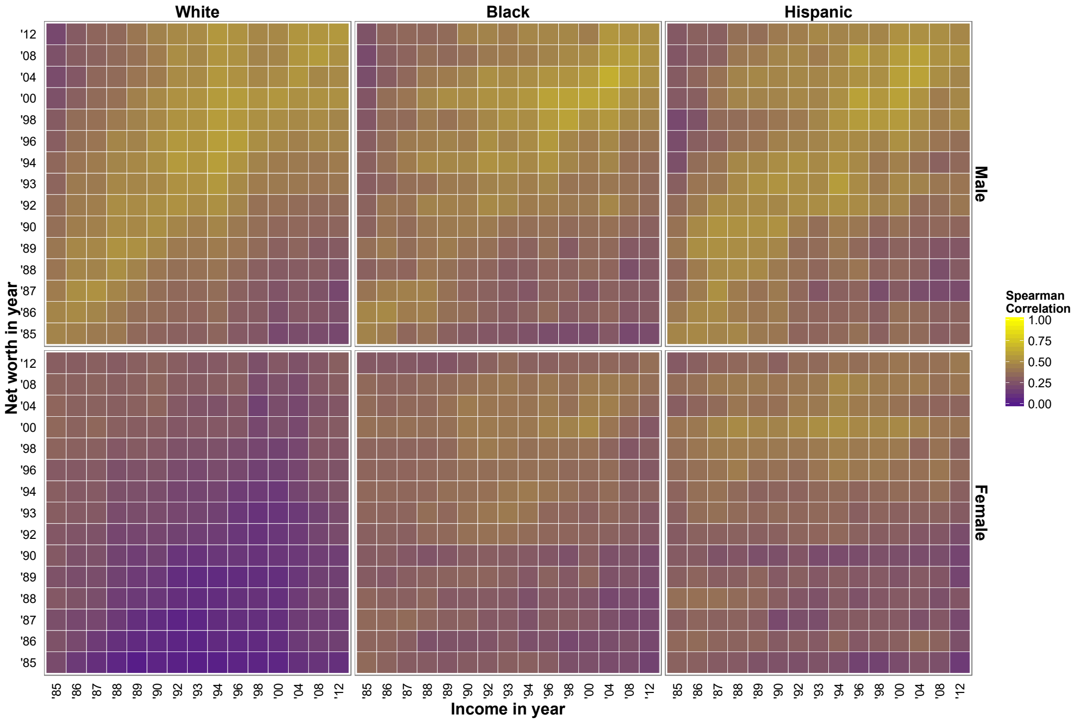

Figure ESM 3. Spearman rank correlations between income and net worth across different waves of the survey (minimum: 0.16; maximum: 0.88).

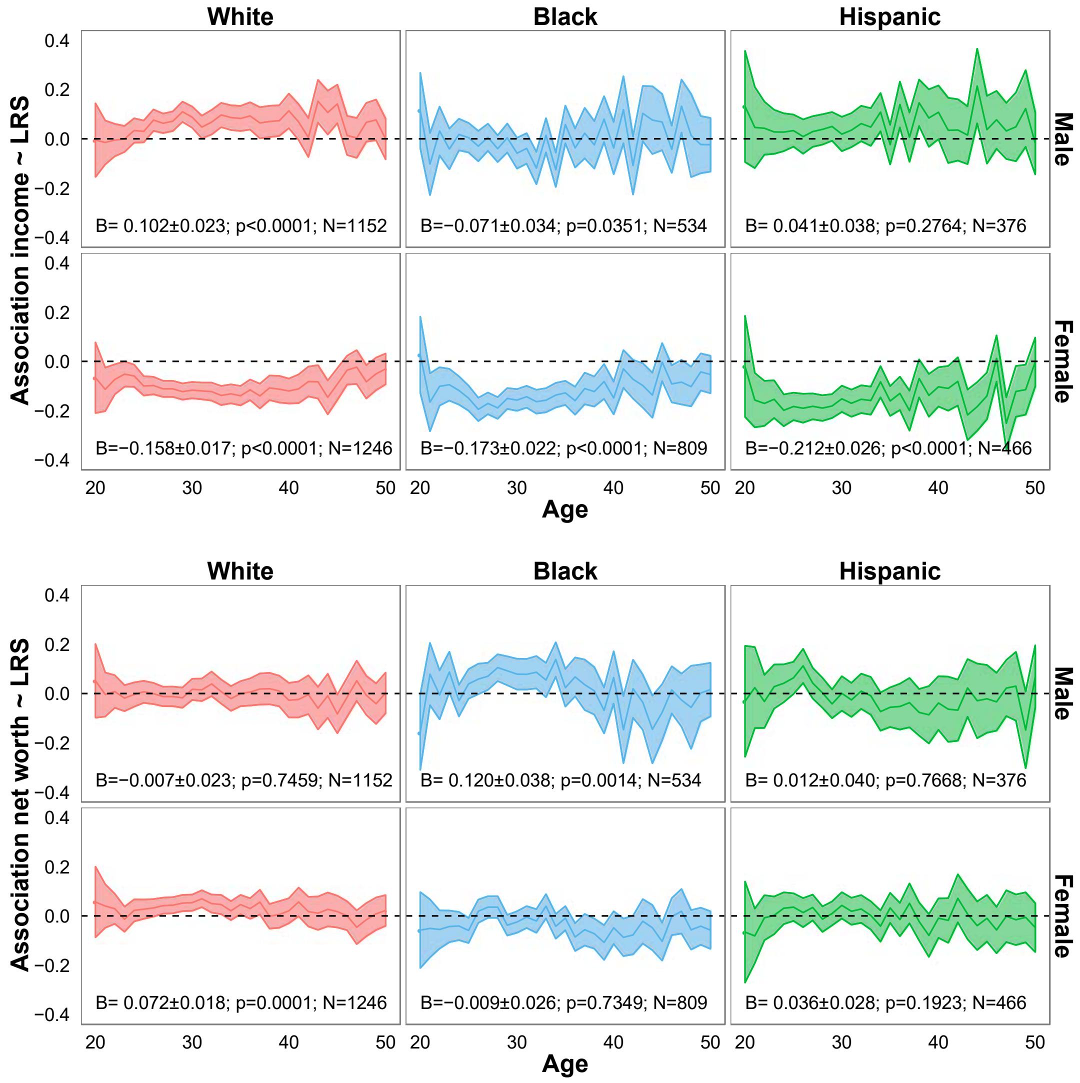

Figure ESM 4. Poisson regression estimates (B; dot) and 95% confidence interval (shaded area) for the effect of income and net worth at a given age and lifetime reproductive success (LRS).

Note that LRS was only determined when the last age at interview exceeded the age of 44. The Poisson estimate (*B*; plus standard error, *p*-value, and sample size) of the effect of an individual's median income or net worth throughout life on LRS is presented at the bottom of each panel. No control variables are included in these models.

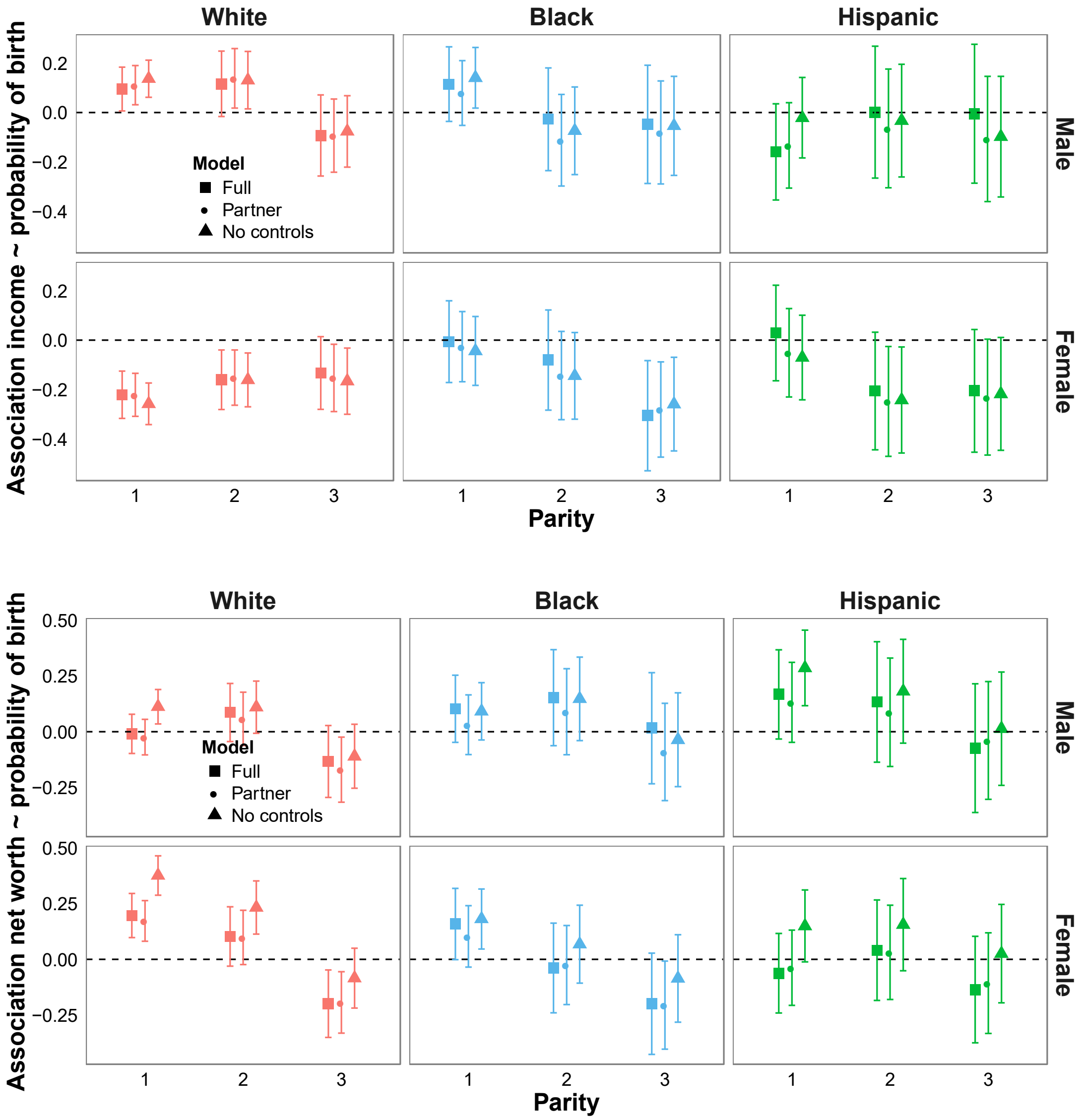

Figure ESM 5. Logistic mixed model parameter estimates (B and 95% confidence interval) of income and net worth measured one year before the time of interview on the probability of a first, second, or third birth (Parity 1, 2, and 3 respectively) within a time period of a year for white, black, and Hispanic men and women.

Interactions between parity and income, and parity and net worth were always included, independent of *p*-value. “No controls” refers to the model that only included the timing variable, parity, their interaction, and excluded relationship status, country of birth, religious affiliations, urban or rural, region of US, maternal education, the number of siblings, and own education. “Partner” refers to the model that only additionally includes relationship status to the “No controls” model. “Full” refers to the model with all control variables. Individual was included as a random intercept. With respect to effect size: e^B represents odds-ratio, with the interpretation that for a randomly chosen individual, the odds of having a birth with a value of wealth of X are e^B times the odds when having a wealth of X-1.

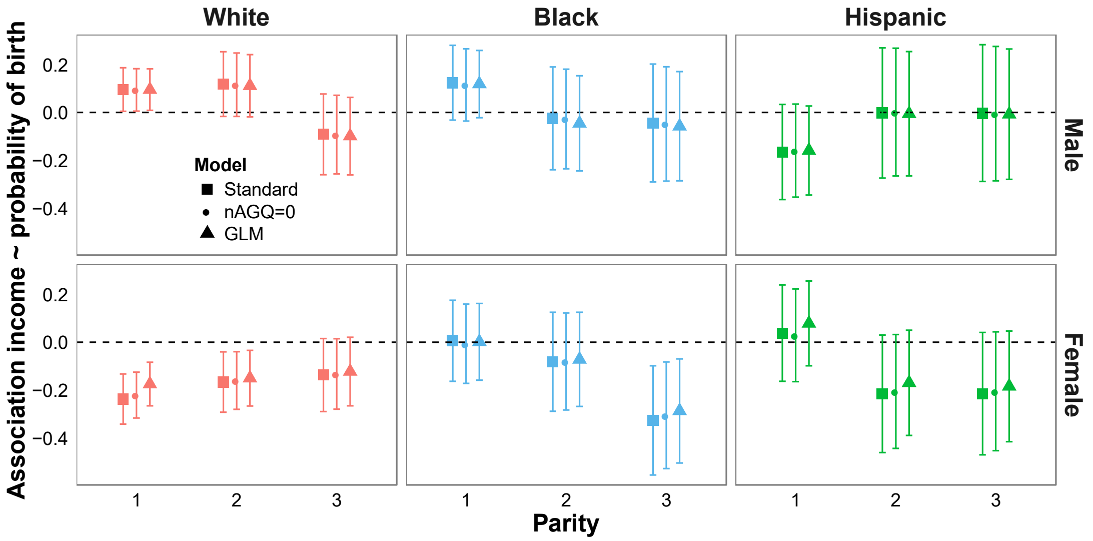

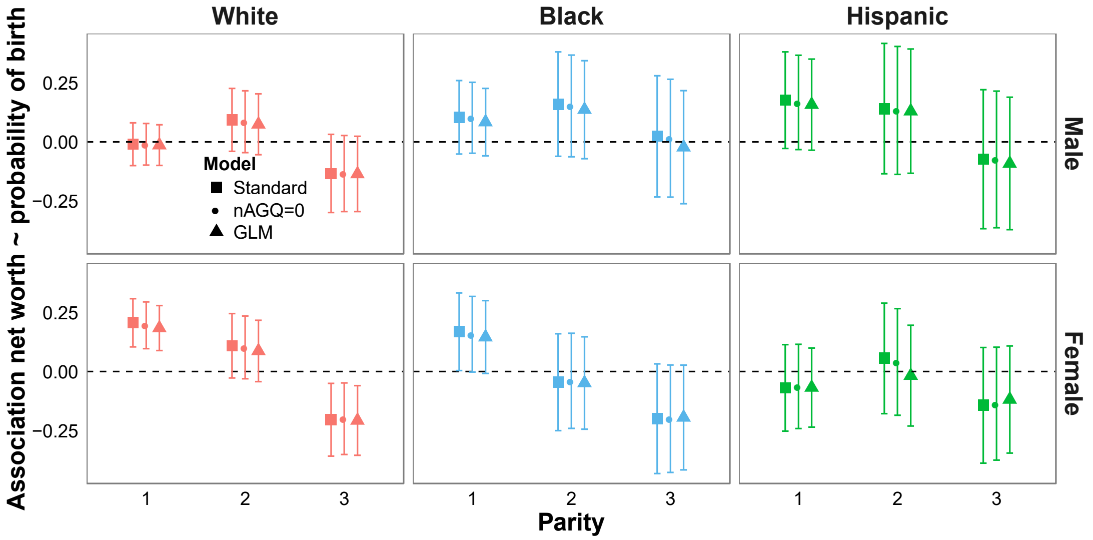

Figure ESM 6. Logistic mixed model parameter estimates (B and 95% confidence interval) of income and net worth measured one year before the time of interview on the probability of a first, second, or third birth (Parity 1, 2, and 3 respectively) within a time period of a year for white, black, and Hispanic men and women.

Interactions between parity and income, and parity and net worth were always included, independent of *p*-value. Individual was included as a random intercept. "Standard" refers to the standard glmer-mixed model run in R (that sometimes did not converge). "nAGQ=0" refers to a glmer-mixed model in which the number of adaptive Gauss-Hermite quadrature points are set to zero. "GLM" refers to a Generalized Linear Model that is similar to the other models, except lacks the random intercept. With respect to effect size: e^B represents odds-ratio, with the interpretation that for a randomly chosen individual, the odds of having a birth with a value of wealth of X are e^B times the odds when having a wealth of X-1.
